# Introduction and validation of the Antisocial Beliefs Scale in a sample of Polish prisoners

**DOI:** 10.3389/fpsyg.2022.991687

**Published:** 2022-10-20

**Authors:** Bartłomiej Skowroński

**Affiliations:** Faculty of Applied Social Sciences and Resocialization, University of Warsaw, Warsaw, Masovian, Poland

**Keywords:** antisocial beliefs, antisocial attitudes, antisocial beliefs scale, attitude, prisoners

## Abstract

**Purpose:**

The goal of this study was to create and validate a brief self-report scale of antisocial beliefs.

**Methods:**

The Antisocial Beliefs Scale (ABS), the Buss–Perry Aggression Questionnaire (BPAQ), the Mach-IV, the IVE Questionnaire were administrated to 718 prisoners. Dimensionality and construct validity of the ABS was investigated using CFA techniques, along with confirmatory bifactor analysis and second-order factor analysis. Four alternatives models of the ABS were specified and tested using Mplus (WLSMV estimation). A comparison sample of adult male non-offenders (n = 339) was also recruited. This sample completed only the ABS.

**Results:**

The bi-factor model offered the best representation of the data. Results suggest that the ABS consists of eight subscales (physical aggression, lack of empathy, absence of prosocial standards, lack of guilt or remorse, incapacity for mutually intimate relationships, risk taking, egocentrism and manipulativeness). The ABS showed different levels of antisocial beliefs in offenders and non-offenders. The factors of ABS correlate significantly with external variables. The Antisocial Beliefs Scale demonstrated very good internal consistency.

**Conclusion:**

The Antisocial Beliefs Scale can be used among participants with criminal history.

## Introduction

### Antisocial beliefs

The most influential model of attitude is the multicomponent model (Rosenberg and Hovland, 1960; Zanna and Rempel, 1988; Eagly and Chaiken, 1993; Haddock and Maio, 2008), in which attitudes are conceptualized as summary evaluations that have affective, cognitive, and behavioral components. Andrews and Bonta define attitudes as follows: “Attitudes are evaluative cognitions and feelings that organize the actor’s decision to act and behaviour toward a person, thing, or action” (Andrews and Bonta, 2010, 234). Attitudes can differ in direction (e.g., positive, neutral, or negative attitudes toward various etnics groups, migrants, etc.) and in strength (e.g., one can feel very strongly or less strongly about a particular issue; Haddock and Maio, 2008).

Among numerous theories and conceptualizations of attitudes, there is one that deserves special attention. In 1978, Polish sociologist and educationalist Czesław Czapów set out the assumptions of his theory of social maladjustment (Czapów, 1978), also known as the social derailment theory (Pytka, 2008). Explaining his concept of attitude, Czapów focused on two components: (1) cognitive beliefs and (2) emotional preferences (Czapów, 1978; Pytka, 2008; Skowroński, 2019). Czapów pointed out the role of beliefs in explaining antisocial behavior based on his concept of attitude. It is worth noting that Czapów publicized his theory at the same time as Beck formulated his cognitive model. Unfortunately, the Polish scholar’s theory did not reach scientists worldwide, because of the Iron Curtain and because the theory was published in Polish.

Criminal or antisocial attitudes are defined as “attitudes/values/beliefs/rationalizations supportive of criminal conduct” (Simourd, 1997, p. 53). Mills and colleagues (Mills et al., 2002) refer to antisocial attitudes as ones related to law, the legal system, criminal others, the police, entitlement, law violations, and violence. Andrews and Bonta identify the components of these attitudes as follows: “antisocial attitudes are thoughts, feelings, and beliefs that are supportive of criminal conduct” (Andrews and Bonta, 2010, p. 234).

One of the first attempts to measure antisocial beliefs was inspired by Beck’s cognitive model (Beck, 1979, 1991; Esposito, 2020), linking the role of cognition to emotional and behavioral elements in the context of depression. According to Beck (Beck, 1991), dysfunctional beliefs impact on the interpretation of internal stimuli (e.g., values) and external stimuli (e.g., events) through cognitive distortions activating dysfunctional emotional reactions and behaviors. Some authors point out that this mechanism seems to be strongest in people with personality disorders (Beck et al., 2015). Esposito (Esposito, 2020, p. 18) states that this is because “their pervasive patterns of thinking and behaving (i.e., same interpretations across a wide range of contexts) and inflexible patterns of thinking and behaving (i.e., rigid interpretations self-serving their beliefs) ultimately lead to a persistent and generalized level of dysfunction” (Esposito, 2020, p. 18).

Antisocial cognition is also one of the factors included in the Central Eight of the Risk–Need–Responsivity Model by Andrews and Bonta. This factor comprises attitudes, values, beliefs, rationalizations, and a personal identity that is favorable to crime (Andrews and Bonta, 2010).

### Antisocial attitudes: Empirical findings

Previous studies conducted among offenders identified the presence of attitudes of loyalty, a tendency to exaggerate society’s shortcomings, self-justification, and belief in luck (Mylonas and Reckless, 1963). Antisocial attitudes are related to neutralizations, which rely on justifying behaviors and include denial of harm to other people, denial of responsibility, a belief that the victims deserve to be hurt, and a belief that the conforming society is an enemy (Sykes and Matza, 1957). Offenders know what is right or wrong, but they have different standards for themselves (Samenow, 1984). Antisocial attitudes comprise criminal rationalizations and negative attitudes towards the law (Simourd and Olver, 2002), high tolerance for deviance, rationalizations for law violations, and a generally antisocial thinking style (Andrews and Bonta, 2010).

The attitudes toward an object are predictive of behavior involving that object (Ajzen and Fishbein, 1977; Ajzen, 2001; Holland et al., 2002; Glasman and Albarracín, 2006), which is why antisocial attitudes have received considerable attention in the prediction of antisocial behavior (Mills et al., 2002). There is evidence in support of the relationship between criminal attitude and criminal behavior, particularly in support of associations between criminal attitude and criminal conduct in individuals belonging to six risk groups (Gendreau et al., 1992; Mills et al., 2002). In another study, adult criminal history, associations with friends involved in criminal behavior, and criminogenic needs—including antisocial attitudes and antisocial personality—were the strongest predictors of recidivism (Gendreau et al., 1995). Moreover, antisocial attitudes were found to be one of the strongest predictors of prison misconduct (Gendreau et al., 1997).

### Assessment of antisocial attitudes

There are different types of assessment models for diagnosing antisociality: the categorical model, which relies on diagnostic criteria to determine the presence or absence of disruptive behaviors (American Psychiatric Association, 2013a), and the dimensional model, referring to a continuum on which a person’s level of abnormal behavior is located (Achenbach, 1991; Achenbach et al., 2005). However, there is growing recognition that categorical assessment (all-or-nothing diagnosis) involves a loss of clinically relevant information and leads to excessive heterogeneity within diagnostic categories (Fowler et al., 2015). The superiority of dimensional models in terms of the predictive and incremental validity of associated personality constructs is supported by a growing number of studies (Samuel and Widiger, 2008; Hopwood and Zanarini, 2010; Morey et al., 2012; Fowler et al., 2015; Ferretti et al., 2021). Additionally, dimensional alternatives offer more reliable and valid approaches to depression (Kotov et al., 2010), well-being (Ozer and Benet-Martinez, 2006), and physical diseases (Deary et al., 2010).

Researchers investigating antisocial/criminal attitudes have used different instruments. The existing measures of antisocial attitudes have different theoretical underpinnings. The Criminal Attribution Inventory (CRAI) by Kroner and Mills (Kroner and Mills, 2008) draws on Sykes and Matza’s concept of neutralization (Sykes and Matza, 1957) and measures criminal responsibility and blame.

The Psychological Inventory of Criminal Thinking Styles by Walters (PICTS) measures cognitive distortions, related to criminal behavior through rationalizations. The theoretical basis of the PICTS is Walters’s criminal lifestyle theory, postulating that this kind of lifestyle is linked to conditions, choices, and cognitions. The thinking errors distinguished by Walters are: Mollification (rationalization of the violation of social norms), Cutoff (denial of own feelings of fear and anxiety), Entitlement (attitudes of ownership and justification), Power Orientation (belief in the control and manipulation of others by the use of aggression), Sentimentality (suppression of guilt by performing good deeds), Superoptimism (belief that one is unsusceptible to the negative consequences of crime), Cognitive Indolence (poor problem solving ability), and Discontinuity (good intentions without good self-discipline; (Egan et al., 2000).

The next measure of cognitive distortions is the How I Think Questionnaire (Barriga and Gibbs, 1996). The theoretical underpinnings of this instrument are provided by the Diagnostic and Statistical Manual (DSM) criteria for oppositional defiant and conduct disorders. The HIT-Q measures stealing, lying, physical aggression, and disrespect for rules, laws, and authority.

The Measures of Criminal Attitudes and Associates (MCAA) was developed by Mills and colleagues (Mills et al., 2002). The criminal attitudes identified by the authors of this measure are: acceptance of violence, attitudes of entitlement, intent to engage in criminal behavior, and identification with and influence of criminal others (Cargill, 2004).

The Antisocial Beliefs and Attitudes Scale (ABAS) by Butler and colleagues (Butler et al., 2015) is a developmentally sensitive measure of young people’s beliefs and attitudes toward social standards of acceptable behavior at home and at school. The ABAS has a three-factor structure comprising Rule Noncompliance, Peer Conflict, and Severe Aggression. Research results provided further support for the ABAS as a reliable and valid measure of antisocial thinking in young people (Butler et al., 2015).

Despite a growing body of research into antisocial attitudes and a growing number of measures, there is a lack of new scales assessing antisocial beliefs based on the antisocial personality disorder criteria. This kind of tool would be useful in offender rehabilitation, which seeks to minimize recidivism by modifying antisocial beliefs (Andrews and Bonta, 2010). Antisocial personality disorder is a predictor of criminal behavior. Moreover, a growing body of studies has revealed that antisocial personality disorder is more strongly related to criminal behavior than any other disorder. ASPD is present in about 47% of incarcerated people (Fazel and Danesh, 2002), in some studies the correlation was even stronger, reaching 78% (Rotter et al., 2002).

The existing instruments have their limitations, too. Some researchers argue that the PICTS is not parsimonious; its scales have been found to be unreliable, and only three of the PICTS the scales are related to criminal behavior (Walters, 1995), which means the measure should be modified to include only those scales that are relevant (Cargill, 2004). Another tool, the CRAI, is limited in its usefulness for predicting criminal behavior, as it measures only criminal thinking and irresponsibility (Cargill, 2004).

There is need to create the new instrument which can be used to assess antisocial beliefs in adults before and after intervention programs (pretest vs. posttest). Antisocial beliefs are one of the eight criminogenic needs which are the target of intervention (Andrews and Bonta, 2010).

### The present study

The aim of my study was to design a new measure that would assess antisocial beliefs. A new reliable and valid tool is necessary to conduct systematic investigations into the cognitive determinants of law-breaking behavior, underpinned by the intent of preventing such behavior.

### Predictions

The criteria for ASPD do not reflect a single dimension but rather are influenced by more than one factor (Kendler et al., 2012). It seems reasonable to expect a multifactor solution as the best conceptualization of the ABS structure. Moreover, it seems reasonable to expect correlations between ABS dimensions and the variables that measure similar constructs: anger, hostility, aggression (physical and verbal), interpersonal tactics, cynical views of human nature, utilitarian morality, impulsivity, venturesomeness and empathy. The positive correlation between ABS dimensions and the external criteria was expected, except the correlation between ABS variables and empathy measured by IVE Questionnaire designed by Eysenck. In this case the negative correlation was expected. The ABS dimensions should correlated significantly with: aggression (Lobbestael et al., 2013; Tackett et al., 2013); impulsiveness (Fossati et al., 2004, 2007; Azevedo et al., 2020) and manipulativeness (Hofer, 1989; Black, 2015).

## Materials and methods

### Sample and procedure

This study was conducted among adult male offenders recruited in eight prisons in Poland (sample 1) and adult men without any criminal record (sample 2). Sample 2 was recruited for comparison purposes only. The two samples as well as recruitment and data collection procedures are described in greater detail below (also see Table 1 for sociodemographic characteristics of both samples).

**Table 1:** Descriptive statistics for demographic variables.

	Prisoners (*N* = 718)	Non-offenders (*N* = 339)
Age in years (*M*/*SD*)	37/10.8	28.6/5.4
Residence (N/%)		
Village	119/16.6	175/51.6
Town (≤50,000)	167/23.3	118/34.8
City (50,000–150,000)	106/14.8	9/2.7
City (>150,000)	311/43.3	37/10.9
Total	703/97.9	339/100
No data	15/2.1	0
Education level (*N*/%)		
Elementary	166/23.1	0
Middle school education	88/12.3	0
Basic vocational	195/27.2	0
Secondary vocational	114/15.9	38/11.2
General secondary	90/12.5	197/58.1
Bachelor’s degree	25/3.5	36/10.6
Master’s degree	25/3.5	68/20.1
Total	703/97.9	339/100
No data	15/2.1	0
Number of sentences (*N*/%)		
1–5	480/66.85	
6–10	155/21.58	
11–15	19/2.64	
16–20	12/1.67	
20<	10/1.39	
Total	676/94.15	
No data	42/5.85	

### Sample 1

I delivered printed anonymous self-report questionnaires to nine prisons administered by the District Inspectorate of the Prison Service in Warsaw (Warsaw Białołęka Detention Center, Warsaw Białołęka Prison, Warsaw Grochów Detention Center, Warsaw Służewiec Detention Center, Radom Detention Center, Grójec Detention Center, Żytkowice Prison, Siedlce Prison, and Płońsk Detention Center). None of the institutions refused to participate in the research. Prior to data collection, the prison personnel were instructed about the purpose of the study and their role in conducting it. The prison staff distributed all questionnaires among prisoners, explained the nature of the study, and provided a summary of informed consent, taking into account that prison inmates were a vulnerable population and that they might feel compelled to take part. It was made clear that participation in the study was voluntary and anonymous. Prisoners were instructed to place completed questionnaires in envelopes and return them to the data collector. Data collection was monitored by the prison staff, and the questionnaires were returned to the author of the study. I approached *N* = 800 prisoners (all of them were male), 752 respondents returned completed surveys (response rate = 89.75%); however, due to significant missing data in some of the questionnaires returned, data from 718 inmates were ultimately included in the analysis. Among prisoners, the oldest participant was born in 1953 and the youngest one in 1999. The largest number of prisoners (456 inmates, 63.5%) had been sentenced under Article 279 of the Polish Penal Code (burglary), 195 prisoners (27.1%) were imprisoned under Article 280 (armed robbery), 32 (4.45%) had committed murder (Article 148 of the Penal Code), 147 (20.47%) were serving a sentence under Article 207 (mistreatment), and 66 (9.19%) were in prison for offenses under Article 286 (fraud, ransom).

### Sample 2

The data of the comparison group (non-offenders) were collected by 36 students, who distributed the questionnaires among adult men without criminal record. The students received appropriate training prior to data collection. Informed consent was obtained from non-offenders as well. The students returned the completed questionnaires to the author of the study. When recruiting the comparison group 36 students reached out to 400 individuals; 366 returned completed surveys, and 27 of them were excluded due to missing data, leaving 339 non-offenders included in the analysis. I included a sample of non-offenders in the study to assess extreme-groups validity. If the ABS is a valid measure of antisocial beliefs, it should yield different scores on this for the two groups (offenders and non-offenders). Among non-offender (comparison) group the oldest respondent was born in 1979 and the youngest one in 1998.

### Measures

The Antisocial Beliefs Scale (ABS) is a 40-item self-report measure designed to assess antisocial beliefs in forensic populations. The ABS consists of eight subscales: (1) Physical Aggression, (2) Lack of Empathy, (3) Absence of Prosocial Standards, (4) Lack of Guilt or Remorse, (5) Incapacity for Mutually Intimate Relationships, (6) Risk Taking, (7) Egocentrism, and (8) Manipulativeness. All responses are indexed on a 4-point Likert scale (1 = *strongly disagree*, 2 = *disagree*, 3 = *agree*, 4 = *strongly agree*).

#### Development of the Antisocial Beliefs Scale

The theoretical basis for the Antisocial Beliefs Scale is the concept of attitude as defined by Czapów (Czapów, 1978), who focused on two components: cognitive beliefs (e.g., “Robbing a dishonest shopkeeper is justified”) and emotional preferences (e.g., “I could rob a dishonest shopkeeper”). Both cognitive beliefs and emotional preferences are internal components of attitude and are related to behavior, which is an external function of attitude (Czapów, 1978; Pytka, 2008; Skowroński, 2019). The author of the Antisocial Beliefs Scale focused on the following criteria: **identity** (egocentrism; self-esteem derived from personal gain, power, or pleasure); **self-direction** (goal-setting based on personal gratification; absence of prosocial internal standards associated with failure to conform to lawful or culturally normative ethical behavior); **empathy** (lack of concern for the feelings, needs, or suffering of others; lack of remorse after hurting or mistreating another); **intimacy** (incapacity for mutually intimate relationships; as exploitation is a primary means of relating to others, including by deceit and coercion; use of dominance or intimidation to control others); **manipulativeness** (frequent use of subterfuge to influence or control others; use of seduction, charm, glibness, or ingratiation to achieve one’s ends); **deceitfulness** (dishonesty and fraudulence; misrepresentation of self; embellishment or fabrication when relating events); **callousness** (lack of concern for feelings or problems of others; lack of guilt or remorse about the negative or harmful effects of one’s actions on others; aggression; sadism); **hostility** (persistent or frequent angry feelings; anger or irritability in response to minor slights and insults; mean, nasty, or vengeful behavior); **irresponsibility** (disregard for—and failure to honor—financial and other obligations or commitments; lack of respect for—and lack of follow through on—agreements and promises); and **risk taking** (engagement in dangerous, risky, and potentially self-damaging activities, unnecessarily and without regard for consequences; boredom proneness and thoughtless initiation of activities to counter boredom; lack of concern for one’s limitations and denial of the reality of personal danger).

The criteria listed above are in line with the DSM–5 alternative model of antisocial personality disorder (ASPD), presented in the “Emerging Measures and Models” chapter (Section III) (American Psychiatric Association, 2013b). Criterion A of the DSM–5 alternative model is focused on impairment in self and interpersonal functioning that is specifically tailored for each personality disorder (American Psychiatric Association, 2013b). In the case of ASPD, impairment in self-functioning is characterized by egocentricity, absence of internal prosocial standards, and failure to conform to lawful behavior; it is also marked by lack of concern for others, lack of remorse, exploitativeness, use of deceit, coercion, dominance, and intimidation to fulfill interpersonal needs (American Psychiatric Association, 2013b; Wygant et al., 2016). Criterion B for personality pathology in the alternative model is focused on the presence of maladaptive personality traits. Accordingly, ASPD is defined by a constellation of manipulativeness, deceitfulness, callousness, and hostility (the antagonism domain) combined with irresponsibility, impulsivity, and risk taking (the disinhibition domain; (American Psychiatric Association, 2013b; Wygant et al., 2016). An ASPD diagnosis requires at least moderate impairment in at least two of the four criterion areas of personality functioning (identity, self-direction, empathy, intimacy) along with elevations on at least six of the seven ASPD-specified traits (Wygant et al., 2016).

Items of the Antisocial Beliefs Scale Items were generated in the course of discussions among a panel of 11 experts in the field of social rehabilitation (psychologists). Based on the criteria listed above, eight dimensions of antisocial beliefs were extracted. The initial list included 224 items rated on 4-point Likert scale (1 = *strongly disagree*, 2 = *disagree*, 3 = *agree*, 4 = *strongly agree*). During the discussions of experts this list was shortened to 120 items. The content validity of the new scale was assessed using Lawshe’s procedure (Lawshe, 1975). As a result, the initial item pool was reduced to 40 items (five per dimension). Scores range from 5 to 20, with higher scores indicating elevated levels of antisocial beliefs.

The Cronbach’s alpha coefficients for the subscales of ABS are: PA = 0.83, LE = 0.80, APS = 0.80, LGR = 0.75, IMIR = 0.75, RT = 0.70, EG = 0.68, MAN = 0.68, and for total score is.94. The ABS had good internal reliability.

The Buss–Perry Aggression Questionnaire (BPAQ) was designed by Arnold H. Buss and Mark Perry and measures four factors: physical aggression, verbal aggression, anger, and hostility. The instrument consists of 29 items rated on a 5-point Likert scale (1 = *extremely uncharacteristic*, 2 = *somewhat uncharacteristic*, 3 = *neither uncharacteristic nor characteristic*, 4 = *somewhat characteristic*, 5 = *extremely characteristic*). The value of Cronbach’s α was.80 for the entire scale, 0.85 for Physical Aggression, 0.72 for Verbal Aggression, 0.83 for Anger, and.77 for Hostility (Aranowska and Rytel, 2012). Scores range from 7 to 35 (angry), from 8 to 40 (hostility), from 5 to 25 (verbal aggression) and from 9 to 45 (physical aggression), with higher scores indicating elevated level of aggression.

The Mach-IV is a three-dimensional 20-item self-report measure of Machiavellianism. Each item is scored on a 7-point Likert scale (1 = *strong disagreement* to 7 = *strong agreement*). The tool measures three theoretically distinguished dimensions: (1) interpersonal tactics, (2) cynical views of human nature, and (3) utilitarian morality. Cronbach’s α for entire scale was.73 (Pilch, 2006). Scores range from 9 to 63 (interpersonal tactics and cynical views of human nature), and from 2 to 14 (utilitarian morality).

The IVE Questionnaire was designed by Eysenck to measure the personality traits of impulsivity, venturesomeness, and empathy. It consists 54 items using a *yes/no* response format. Cronbach’s α for impulsivity was 0.86 for women and 0.76 for men, for venturesomeness it was 0.90 for women and.85 for men, and for Empathy it was.77 for both genders (Jaworowska, 2011). Scores range from 0 to 19 (impulsivity), from 0 to 16 (venturesomeness), and from 0 to 19 (empathy).

The ABS was completed by prisoners and comparison group, and The Buss–Perry Aggression Questionnaire, The Mach-IV, and IVE Questionnaire were completed by prisoners group only.

### Analytical procedure

I performed confirmatory factor analysis (CFA) to test the following models: a model with one latent factor (M1), a multifactor model (M2), a second-order model (M3), and a bifactor model (M4). The author of the social derailment theory did not strictly define the structure of antisocial beliefs. This made it necessary to test different models of their structure (one-factor, second-order, bifactor, and multifactor models). CFA was only performed with the prisoner sample.

Kline (Kline, 2015) suggested that model fit should be estimated using at least four indicators: two absolute fit indices: (1) RMSEA (root mean square error of approximation) and (2) SRMR (standardized root mean square residual; (Steiger, 1990; Kline, 2015), and two relative fit indices: (1) TLI (Tucker–Lewis index) and (2) CFI (comparative fit index); (Bentler, 1990; Kline, 2015). The indicators listed were used to test which model was the best fit to the data set. An adequately fitting model should have CFI and TLI values higher than or equal to 0.95 (Yu, 2002), and RMSEA should be close to 0.06 (Hu and Bentler, 1999), but Kline (Kline, 2015) suggested that the CFI value should be higher than or equal to.90 and that the TLI should be higher than.95. The cut-off for SRMR indicating good fit is.08 (Kline, 2015). All calculations were carried out using Mplus 8.2 with WLSMV estimation (Muthén and Muthén, 2012). Descriptive statistics, the Mann-Whitney U test, were calculated using SPSS version 28. Criterion validity for the ABS was assessed using a series of pairwise correlation coefficients calculated in SPSS. The estimation of the reliability of ABS was provided by examining the composite reliability using the following formula (Netemeyer et al., 2003):


$$\frac{{\left(\Sigma_{i=1}^{p}\lambda_{i}\right)}^{2}}{{\left(\Sigma_{i=1}^{p}\lambda_{i}\right)}^{2}+\Sigma_{i}^{p}V\left(\delta \right)}$$

λ_*i*_ = completely standardized loading for the *i*th indicator,

*V*(δ_*i*_) = variance of the error term for the *i*th indicator,

*p* = number of indicators.

## Results

### Descriptive statistics

Descriptive statistics for eight factors representing antisocial beliefs: physical aggression (PA), lack of empathy (LE), absence of prosocial standards (APS), lack of guilt or remorse (LGR), incapacity for mutually intimate relationships (IMIR), risk taking (RT), egocentrism (EG), and manipulativeness (MAN) and BPAQ, MACH-IV and IVE are presented in Tables 2, 3.

**Table 2:** Descriptive statistics for ABS factors: prisoners (*N* = 718); non-offenders (*N* = 339).

Variables	Prisoners						Non-offenders					
	*M*	*SD*	Min.	Max.	Skewness	Kurtosis	*M*	*SD*	Min.	Max.	Skewness	Kurtosis
Physical aggression (PA)	10.66	4.55	5	25	0.528	−0.367	7.25	1.55	5	11	0.245	−0.481
Lack of empathy (LE)	10.97	4.25	5	22	0.331	−0.626	8.86	2.53	5	15	0.509	−0.296
Absence of prosocial standards (APS)	11.58	4.53	5	25	0.123	−0.815	6.97	1.87	5	12	0.962	0.264
Lack of guilt or remorse (LGR)	11.00	4.06	5	24	0.313	−0.440	6.6	1.67	5	12	1.183	1.371
Incapacity for mutually intimate relationships (IMIR)	10.81	4.24	5	25	0.490	−0.211	8.36	2.28	5	13	0.108	−0.993
Risk taking (RT)	12.55	4.25	5	25	0.027	−0.446	10.12	3.18	5	17	0.250	−0.826
Egocentrism (EG)	11.81	4.01	5	25	0.249	−0.195	8.18	1.94	5	12	0.144	−0.854
Manipulativeness (MAN)	12.41	4.02	5	24	−0.041	−0.446	8.22	2.31	5	13	0.290	−0.952
Antisocial beliefs: total score	91.77	27.91	40	185	0.85	−0.98	64.59	13.82	42	94	0.17	−0.98

**Table 3:** Descriptive statistics for BPAQ, MACH-IV, and IVE for prisoners group.

	Variables	*M*	*SD*	Min.	Max.	Skewness	Kurtosis
BPAQ	Anger	18.82	5.29	7	33	0.228	−0.460
	Hostility	22.35	6.38	8	40	0.049	−0.191
	Physical aggression	22	6.79	9	45	0.391	−0.230
	Verbal aggression	15.04	3.80	5	25	−0.034	−0.034
	BPQA global score	78.22	18.97	35	136	0.188	0.188
MACH-IV	Interpersonal tactics	31.93	7.15	9	52	−0.101	0.023
	Cynical views of human nature	34.31	6.32	9	57	−0.336	1.474
	Utilitarian morality	7.98	2.57	2	14	−0.172	0.626
IVE	Impulsivity	9.72	4.56	1	19	0.041	−0.870
	Venturesomeness	9.57	2.85	2	16	−0.168	−0.567
	Empathy	10.34	2.65	3	19	0.302	0.087

BPAQ, The Buss–Perry Aggression Questionnaire; MACH-IV, Machiavellianism Test; IVE, Eysenck’s Impulsivity Inventory.

### Confirmatory factor analysis results and correlations between ABS dimensions

The fit indices of the four models tested for items measuring antisocial beliefs are shown in Table 4. The analysis of the values of fit indices shows that all of them (RMSEA, CFI, TLI, and SRMR) indicate adequate fit of the tested models to the data.

**Table 4:** Fit indices for alternative measurement models for the Antisocial Beliefs Scale.

Model	*χ*^2^ (*df*)	CFI	TLI	SRMR	RMSEA	RMSEA 90% CI
M1: ONE-FACTOR	3232.162 (740)*	0.920	0.916	0.055	0.068	[0.066, 0.071]
M2: MULTIFACTOR	2696.983 (712)*	0.934	0.929	0.052	0.062	[0.060, 0.065]
M3: SECOND-ORDER	2781.919 (732)*	0.935	0.930	0.051	0.062	[0.060, 0.065]
M4: BIFACTOR	2462.962 (700)*	0.944	0.937	0.047	0.059	[0.057, 0.062]

*p < 0.001.

**Figure 1:**
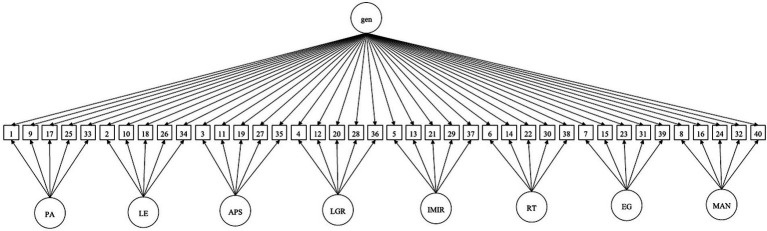
Bi-factor model of the Antisocial Beliefs Scale. PA, physical aggression; LE, lack of empathy; APS, absence of prosocial standards; LGR, lack of guilt or remorse; IMIR, incapacity for mutually intimate relationships; RT, risk taking; EG, egocentrism; MAN, manipulativeness.

Although the one-factor model is acceptable, its values of fit are slightly lower than those of the remaining models. The results presented in Table 4 indicate that the bifactor model reflects the best conceptualization of the data. The bifactor model incorporates a general factor, which loads directly on all observed variables in the model, and grouping factors, which load on subgroups of the same set of observed variables (Dunn and McCray, 2020). The factor loadings are lower for grouping factors, at the same time they are higher for general factor. This indicates that the ABS is rather unidimensional measure. A visual scheme of the bi-factor model of the ABS is presented in Figure 1.

The standardized factor loadings for the general factor and the eight dimensions of antisocial beliefs are provided in Table 5.

**Table 5:** Standardized factor loadings for the general factor and the factors representing eight antisocial beliefs.

Original items numbers	PA	LE	APS	LGR	IMIR	RT	EG	MAN	General factor
Inducing a sense of inferiority in others gives a sense of satisfaction.	0.417*								0.764*
It does not matter what other people feel.		−0.045*							0.763*
It is not particularly bad to rob someone rich.			0.402*						0.716*
Apologizing is a sign of weakness.				0.097					0.728*
Maintaining a relationship with a woman is an tiresome duty.					0.409*				0.695*
Fast driving is not dangerous at all.						0.223*			0.507*
You should not share what you have with others in a disinterested way.							0.177*		0.556*
Doing things to spite others is an interesting game.								−0.044*	0.685*
Fist fighting is one of the most effective ways of resolving conflicts.	0.204*								0.740*
It is not worth letting others jump the checkout line, even if they need it.		0.095*							0.738*
There is nothing wrong with petty theft.			0.316*						0.719*
Only weak people have a sense of guilt.				0.231*					0.685*
Women are mostly too bossy and unsympathetic.					0.178*				0.634*
Life without risk would be monotonous.						0.608*			0.508*
Everyone should earn money only for themselves.							0.153*		0.587*
It can be exciting to provoke policemen with your behavior.								0.129*	0.682*
Animals should be physically punished for their misbehavior.	0.251*								0.695*
People who cry are irritating.		0.239*							0.702*
There is nothing wrong with sometimes stealing things that aren’t of much value.			0.392*						0.686*
There is no point in thinking about your behavior too much.				0.119*					0.712*
It is humiliating for a man to seek popularity with women.					0.435*				0.624*
Contrary to popular belief, high board diving is always safe.						0.060*			0.554*
In life you have to think mainly about yourself.							0.381*		0.472*
Sometimes when asking someone for help it is better not to give the real reasons for your request.								0.333*	0.523*
A man who does not want to fight is not a real man.	0.094*								0.736*
Approaching people lying on the pavement to help them can bring nothing but problems.		−0.067*							0.718*
Only a written agreement between parties is valid.			−0.137*						0.673*
Remorse is a sign of weakness.				0.301*					0.695*
It is actually hard to say what it means to “be in love” with a woman.					0.183*				0.607*
Hitchhiking at night is exciting.						0.330*			0.475*
It only pays to do what brings real benefit.							0.507*		0.612*
In some situations you have to lie to get out of trouble.								0.589*	0.339*
It is normal for people to degrade others.	0.366*								0.711*
One should not bother about other people’s problems.		0.443*							0.720*
Stealing something from a store owned by a dishonest shopkeeper is not a bad thing.			0.374*						0.621*
Hitting another person as a result of annoyance can be partially justified.				−0.369*					0.638*
One should avoid relationships with women should be avoided.					0.409*				0.546*
Life without adrenaline is boring.						0.587*			0.512*
Everything we do stems from the desire to satisfy only our own needs.							0.209*		0.579*
Provoking a quarrel can give considerable satisfaction.								−0.086*	0.704*

PA, physical aggression; LA, lack of empathy; APS, absence of prosocial standards; LGR, lack of guilt or remorse; IMIR, incapacity for mutually intimate relationships; RT, risk taking; EG, egocentrism; MAN, manipulativeness.

### Sample comparisons

The differences in all variables measured by the Antisocial Beliefs Scale between the group of prisoners and the group of men who had never been convicted are statistically significant at *p* < 0.001. The means were significantly higher for prisoners than for comparison group (non-offenders). The details are shown in Table 6. The Antisocial Beliefs Scale shows higher level of antisocial beliefs in offenders group comparing with non-offenders.

**Table 6:** Differences between prisoners and non-offenders in ABS dimensions.

	*M*_rank_		*U*	*Z*
	Prisoners (*N* = 718)	Non-offenders (*N* = 339)		
Physical aggression (PA)	601.73	374.96	69,481.5	−11.34*
Lack of empathy (LE)	576.74	427.89	87,426	−7.42*
Absence of prosocial standards (APS)	627.17	321.08	51,216.5	−15.29*
Lack of guilt or remorse (LGR)	638.01	298.11	43,429.5	−17.01*
Incapacity for mutually intimate relationships (IMIR)	585.53	409.28	81,115.5	−8.8*
Risk taking (RT)	587.17	405.79	79,934.5	−9.04*
Egocentrism (EG)	623.85	328.10	53,595.5	−14.75*
Manipulativeness (MAN)	632.72	309.32	47,230.5	−16.12*
Antisocial beliefs: total score	626.86	321.73	51,435.5	−15.17*

*p < 0.001.

All eight factors were found to be intercorrelated (see Table 7). Most of the correlations, however, were not as high as to indicate that they measured the same phenomenon. Only four correlations were above.70 (high and positive). Most of correlations were moderate and positive or even low and positive, as the one between risk taking and incapacity for mutually intimate relationships.

**Table 7:** Correlations between ABS factors.

Factor	PA	LE	APS	LGR	IMIR	RT	EG	MAN	ABTS
Physical aggression (PA)	1								
Lack of empathy (LE)	0.744^**^	1							
Absence of prosocial standards (APS)	0.699^**^	0.698^**^	1						
Lack of guilt or remorse (LGR)	0.740^**^	0.789^**^	0.717^**^	1					
Incapacity for mutually intimate relationships (IMIR)	0.683^**^	0.696^**^	0.621^**^	0.650^**^	1				
Risk taking (RT)	0.519^**^	0.537^**^	0.599^**^	0.601^**^	0.456^**^	1			
Egocentrism (EG)	0.559^**^	0.659^**^	0.594^**^	0.623^**^	0.580^**^	0.501^**^	1		
Manipulativeness (MAN)	0.631^**^	0.646^**^	0.694^**^	0.673^**^	0.546^**^	0.584^**^	0.604^**^	1	
Antisocial beliefs: total score (ABTS)	0.856^**^	0.871^**^	0.885^**^	0.889^**^	0.811^**^	0.764^**^	0.821^**^	0.849^**^	1

**p < 0.001.

### Criterion validity

Table 8 shows associations between the eight ABS factors and external variables. Physical aggression (PA) correlated positively with BPAQ total score, anger, hostility, physical and verbal aggression. As expected, lack of empathy (LE) correlated negatively with empathy (IVE). Absence of prosocial standards (APS) and lack of guilt or remorse (LGR) correlated positively with utilitarian morality. The next factor, incapacity for mutually intimate relationships, correlated positively with impulsivity, BPQA: total score and interpersonal tactics. As predicted, risk taking was most strongly positively correlated with venturesomeness and impulsivity. Moreover, egocentrism was a negative predictor of empathy, while manipulativeness correlated positively with aggression (physical and verbal), anger, hostility, BPQA: total score, the number of convictions, and utilitarian morality.

**Table 8:** Associations between the eight ABS factors and external variables.

	BPQA					MACH-IV			IVE			Number of convictions
	Anger	Hostility	Physical aggression	Verbal aggression	BPQA: total score	Interpersonal tactics	Cynical views of human nature	Utilitarian morality	Impulsivity	Venturesomeness	Empathy	
Physical aggression	0.269**	0.277**	0.397**	0.195**	0.350**	0.268**	0.086*	0.088*	0.389**	0.177**	−0.220**	n.s.
Lack of empathy	0.187**	0.223**	0.332**	0.125**	0.271**	0.290**	0.158**	0.164**	0.336**	0.156**	−0.305**	n.s.
Absence of prosocial standards	0.233**	0.257**	0.364**	0.175**	0.317**	0.306**	0.171**	0.209**	0.423**	0.239**	−0.193**	n.s.
Lack of sense of guilt and remorse	0.263**	0.262**	0.400**	0.165**	0.338**	0.308**	0.168**	0.200**	0.399**	0.190**	−0.229**	n.s.
Incapacity for mutually intimate relationships	0.178**	0.198**	0.269**	n.s.	0.227**	0.224**	0.146**	0.050*	0.326**	n.s.	−0.193**	0.084*
Risk taking	0.273**	0.251**	0.307**	0.221**	0.315**	0.258**	n.s.	0.149**	0.422**	0.386**	−0.074*	n.s.
Egocentrism	0.265**	0.213**	0.316**	0.182**	0.295**	0.248**	0.205**	0.188**	0.356**	0.196**	−0.240**	n.s.
Manipulativeness	0.306**	0.353**	0.392**	0.252**	0.395**	0.262**	0.189**	0.232**	0.424**	0.271**	−0.122**	0.101**
Antisocial beliefs: total score	0.299**	0.309**	0.422**	0.210**	0.381**	0.329**	0.176**	0.193**	0.467**	0.254**	−0.240	n.s.

**p < 0.01; *p < 0.05.

### Internal reliability

I computed composite reliability to determine the internal reliability of the tool. The ABS had good internal reliability (0.966).

## Discussion

The Antisocial Beliefs Scale is a promising alternative to the existing measures of antisocial attitudes. What served as the theoretical basis for the ABS was the criteria of the DSM-5 alternative model of ASPD, presented in the “Emerging Measures and Models” chapter (Section III); (American Psychiatric Association, 2013b), and Czapów’s social derailment theory (Czapów, 1978). According to Czapów, beliefs and preferences are internal functions of attitudes. The Antisocial Beliefs Scale measures the former component of antisocial attitude.

In a critical evaluation of psychopathy measurement, Boduszek and Dębowska state that it is unacceptable to assume that only one model exists for a particular measure and that, in order to explore the dimensionality of the measure, it is advisable to test competing solutions (Boduszek and Dębowska, 2016). Following this recommendation, in the process of ABS validation I tested four competing models: one-factor, multi-factor, second-order, and bifactor models. Generally, all models offered acceptable fit to the data (assessed using CFI, TLI, RMSEA, and SRMR), but the best-fitting one was the bifactor model. However, the factor loadings are lower for grouping factors, than for general factor. It indicates that the ABS is unidimensional rather than multidimensional measure. Theory of social maladjustment linking the role of antisocial beliefs in the context of antisocial behavior (Czapów, 1978). There is still no consensus on the nature and dimensionality of antisocial behavior (dos Santos et al., 2019). Some authors consider this construct to be a syndrome of problem behavior defined by one factor (Jessor et al., 1991; Farrington, 1995; Farrell et al., 2000). In turn, an alternative point of view suggests that antisocial behavior presents a multidimensional nature (Loeber and Stouthamer-Loeber, 1998; Thornberry and Loeber, 2004; dos Santos et al., 2019; Burke et al., 2022).

The correlations between the factors of the Antisocial Beliefs Scale were mainly moderate and positive, but four correlations were high and positive (above.70): between physical aggression and lack of empathy and between lack of guilt or remorse and three factors: physical aggression, absence of prosocial standards, and lack of guilt or remorse. If the latent factors are highly intercorrelated, additional tests are needed to verify if these factors correlate differently with external variables (Carmines and Zeller, 1979; Boduszek and Dębowska, 2016). The correlations between the ABS dimensions and external variables confirm that the specified factors correlate differently with external variables. On the other hand, the moderate to strong intercorrelations among the dimensions of antisocial attitudes are not unexpected (Mills et al., 2002). Millar and Tesser (1986) found that stronger correlations among an attitude’s dimensions were associated with increased polarization of the attitude.

The Antisocial Beliefs Scale showed different levels of antisocial beliefs in offenders and non-offenders. The hypothesized difference in antisocial beliefs is reflected in the scores of these groups.

The reliability of the Antisocial Beliefs Scale was assessed. Cronbach’s α for the following dimensions: physical aggression, lack of empathy, absence of prosocial standards; lack of guilt or remorse, incapacity for mutually intimate relationships and risk taking ranged between 0.702 and.830 indicating very good internal consistency and for egocentrism and manipulativeness were slightly lower than.70 indicating acceptable internal consistency.

The presented instrument can be used to assess antisocial beliefs in adults before and after intervention programs (pretest vs. posttest). The antisocial beliefs measured by the Antisocial Beliefs Scale correspond to antisocial cognitions (Andrews and Bonta, 2010), which constitute one of the dynamic criminogenic needs (Gendreau et al., 1992) addressed by interventions through delivering human services in accordance with the principles of the Risk–Need–Responsivity model. Antisocial beliefs are considered a dynamic factor in the prediction of risk (Mills et al., 2002).

Individuals with ASPD were more prone to recidivism (Bonta et al., 1998). The Antisocial Beliefs Scale, based on ASPD criteria, is a promising and valid measure of antisocial attitude.

The study has several methodological weaknesses. The analysis was based on data from a Polish prison population, and the findings cannot be generalized to other groups. Future studies should take different linguistic and cultural contexts into account. The next limitation is the cross-sectional design, making it impossible to determine the causal relationships between ABS factors and external criteria. A longitudinal design should be used in future research. There is strong evidence supporting the conclusion that behavior can be predicted by attitudes (Ajzen and Fishbein, 1977; Ajzen, 2001; Holland et al., 2002; Glasman and Albarracín, 2006). In this light, the antisocial beliefs measured by the Antisocial Beliefs Scale can be considered as a potential predictor of antisocial behavior (Gendreau et al., 1992, 1995, 1997; Mills et al., 2002) in future longitudinal research. It would be interesting to test if the variables measured by the Antisocial Beliefs Scale predict recidivism, time spent in prison, or different times of offences. Another limitation is the substantial demographic differences between the two samples. This applies both to the level of education (much lower in prisoners) and to place of residence (approx. 43% of prisoners had lived in cities with 150,000 inhabitants or more before imprisonment).

## Data availability statement

The raw data supporting the conclusions of this article will be made available by the authors, without undue reservation.

## Ethics statement

The studies involving human participants were reviewed and approved by Bioethics Committee of the Institute of Social Prevention and Resocialization, University of Warsaw. The patients/participants provided their written informed consent to participate in this study.

## Author contributions

The author confirms being the sole contributor of this work and has approved it for publication.

## Conflict of interest

The author declares that the research was conducted in the absence of any commercial or financial relationships that could be construed as a potential conflict of interest.

## Publisher’s note

All claims expressed in this article are solely those of the authors and do not necessarily represent those of their affiliated organizations, or those of the publisher, the editors and the reviewers. Any product that may be evaluated in this article, or claim that may be made by its manufacturer, is not guaranteed or endorsed by the publisher.
